# 20-Hydroxyecdysone (20E) signaling as a promising target for the chemical control of malaria vectors

**DOI:** 10.1186/s13071-020-04558-5

**Published:** 2021-01-29

**Authors:** Elodie Ekoka, Surina Maharaj, Luisa Nardini, Yael Dahan-Moss, Lizette L. Koekemoer

**Affiliations:** 1 WITS Research Institute for Malaria, School of Pathology, Faculty of Health Sciences, University of the Witwatersrand, Johannesburg, South Africa; 2 Centre for Emerging, Zoonotic & Parasitic Diseases, National Institute for Communicable Diseases, Johannesburg, South Africa

**Keywords:** Steroid hormone, Chemical control, 20E agonist, 20E antagonist, Insecticide resistance, Synergists, Vector abundance, Vector competence

## Abstract

With the rapid development and spread of resistance to insecticides among anopheline malaria vectors, the efficacy of current World Health Organization (WHO)-approved insecticides targeting these vectors is under threat. This has led to the development of novel interventions, including improved and enhanced insecticide formulations with new targets or synergists or with added sterilants and/or antimalarials, among others. To date, several studies in mosquitoes have revealed that the 20-hydroxyecdysone (20E) signaling pathway regulates both vector abundance and competence, two parameters that influence malaria transmission. Therefore, insecticides which target 20E signaling (e.g. methoxyfenozide and halofenozide) may be an asset for malaria vector control. While such insecticides are already commercially available for lepidopteran and coleopteran pests, they still need to be approved by the WHO for malaria vector control programs. Until recently, chemicals targeting 20E signaling were considered to be insect growth regulators, and their effect was mostly studied against immature mosquito stages. However, in the last few years, promising results have been obtained by applying methoxyfenozide or halofenozide (two compounds that boost 20E signaling) to *Anopheles* populations at different phases of their life-cycle. In addition, preliminary studies suggest that methoxyfenozide resistance is unstable, causing the insects substantial fitness costs, thereby potentially circumventing one of the biggest challenges faced by current vector control efforts. In this review, we first describe the 20E signaling pathway in mosquitoes and then summarize the mechanisms whereby 20E signaling regulates the physiological processes associated with vector competence and vector abundance. Finally, we discuss the potential of using chemicals targeting 20E signaling to control malaria vectors.

**Graphical abstract:**

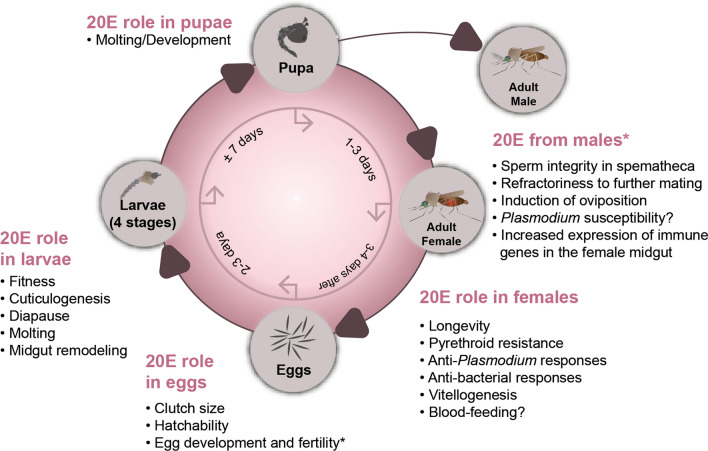

## Background

Malaria is spread when *Plasmodium* parasites are transmitted between humans* via* hematophagous female anopheline mosquitoes. While the 2019 statistics (409,000 deaths globally and ± 3.0 billion USD invested in malaria control and elimination programs) already reveal the high socio-economic impact of this disease [1], recent models predict that with the unprecedented coronavirus disease 2019 (COVID-19) pandemic, deaths due to malaria in low- and middle-income countries could increase by 36% over the next 5 years [2]. Therefore, African countries, where currently more than 90% of all malaria deaths worldwide occur [1], are the most at risk.

The burden of malaria is managed by a multi-disciplinary approach which combines targeting the parasite (artemisinin combination therapy) [3, 4], the vector (World Health Organization [WHO]-approved insecticides and long-lasting insecticide treated bednets) [5, 6] and, to some extent, the environment (habitat modification or larval source management) [7–9]. Additionally, two vaccines are currently under trial: RTS,S and AGS-v [10–13]. The pre-erythrocytic stage antimalarial vaccine RTS,S targets the circumsporozoite surface protein of *Plasmodium falciparum* and is currently in phase IV clinical trials in Ghana, Kenya, and Malawi [1, 11, 12]. In contrast, the AGS-v vaccine targets four conserved saliva peptides in *Anopheles* spp., *Aedes* spp. and *Culex* spp., and has shown promising results in terms of safety and immunogenicity during its phase I clinical trial in humans [10]. Of all these interventions, vector control plays a central role. In fact, the WHO has stated that “vector control is a vital component of malaria prevention, control, and elimination strategies because it can be highly effective in providing personal protection and/or reducing disease transmission” [6].

The WHO-approved vector control strategies can be divided into two categories, referred to as core interventions and supplementary interventions [6]. Core interventions comprise incorporating insecticides into bednets (long-lasting insecticide-treated nets [LLINs]) or spraying insecticides onto the walls of houses (indoor residual spraying [IRS]). At present, four WHO-approved classes of insecticides are used in IRS interventions, namely pyrethroids, carbamates, organophosphates and organochlorines, as opposed to LLINs for which only pyrethroids have been approved owing to their relative safety [6]. Collectively, LLINs and IRS have led to an 18% global reduction in malaria cases over the past 8 years [14]. Supplementary interventions, on the other hand, include larval source management (LSM)* via* biological or chemical larvicides, as well as the disruption of breeding sites [6]. Although there are reports of highly successful LSM programs [15], in reality most malaria-endemic countries have so many breeding sites that LSM becomes both expensive and impractical.

Unfortunately, field resistance to insecticides is common and widespread, with the result that the efficacy of the core interventions has been drastically impaired. In particular, pyrethroid resistance has been detected in all the major African malaria vectors, including *Anopheles gambiae*, *An. coluzzii*, *An*. *arabiensis* and *An*. *funestus* [16, 17]. This has created an urgent need for enhanced insecticides, such as those carrying synergists (e.g. piperonyl butoxide) [18–20], antimalarials (e.g. atovaquone) [21] or chemicals with novel targets. The latter would ideally target a mosquito pathway that is essential for vector competence and vector abundance, have minimal effect on non-target species and be effective against mosquitoes that are resistant to the classes of insecticides currently under use. One such pathway of interest is that of the steroid hormone, 20-hydroxyecdysone (20E) [22]. Indeed, studies in *An. gambiae* suggest that chemicals which target the 20E signaling pathway have the potential to control malaria vectors, both at the adult [23–25] and immature stages [26, 27]. This is because the 20E pathway regulates several key physiological processes in mosquitoes, such as blood-feeding, insecticide resistance, pathogen development, molting, mating, fecundity and fertility (Table 1). In this review, we first describe the 20E signaling pathway in mosquitoes, then discuss the mechanisms whereby 20E signaling regulates the physiological processes associated with vectorial capacity, such as susceptibility to *Plasmodium* infection, egg production and development. Finally, we discuss the potential of chemical control interventions targeting 20E signaling to reduce the burden of malaria.

**Table 1:** Phenotypes associated with manipulating 20-hydroxyecdysone titers, activity or signaling in mosquitoes

Method	Species	Phenotype	References
***20E signaling regulates egg production and egg hatching***			
20E injection (engorged females)	*An. freeborni*	Longer retention of blood meal	[171]
20E injection (virgin females)	*An. arabiensis*,* An. gambiae*	Increased oviposition and refractoriness to further mating	[122]
EcR silencing (adult females)	*Ae. aegypti*	Reduced egg production Inhibition genes involved in autophagy Decreased size of ovarian follicles Egg developmental defects (failure of eggs to develop after first oviposition)	[172]
EcR silencing (adult females)	*An. gambiae*	~ 74.4% decreased expression of *MISO* (gene regulating oogenesis and oviposition) ~ 54% decreased expression of *Vg* (gene regulating vitellogenesis)	[123]
EcR silencing (adult females)	*An. gambiae*	Reduced egg clutch size	[105]
USP silencing (adult females)	*An. gambiae*	Reduced correlation between egg production and pathogen development	[105]
20E agonist methoxyfenozide (eggs)	*Cx. pipiens*	46.99% inhibitory effect on egg hatchability Slowed development Atypical hatching observed	[131]
20E agonist halofenozide (4th instar larvae)	*Cx. pipiens*	Developmental abnormalities in newly eclose adults 23% reduction of hatch rate and 14% reduction of fecundity	[173]
20E agonist methoxyfenozide (adult females)	*An. gambiae*	95% reduction in egg batch size 98.7% of treated females lacked mature ovarian follicles	[23]
Reducing 20E titers and activity (adult males)	*An. gambiae*	Females mating with those 20E-impaired males fail to oviposit after blood feeding	[25]
Reducing 20E activity (adult females)	*An. gambiae*	Reduced egg clutch size	[105]
***20E signaling regulates larval/pupal development***			
20E agonist halofenozide (4th instar larvae)	*Cx. pipiens*	Developmental abnormalities observed in larvae and pupae Decreased body weight of fourth instar larvae due to decreased nutrient uptake	[173]
20E agonists RH-5849, RH-5992 & RH-2485 (larvae)	*Ae. aegypti*,* Cx. quinquefasciatus*, *An. gambiae*	Concentrations above 100 μg/100 ml resulted in 100% larval mortality in *An. gambiae* Concentrations above 200 μg/100 ml resulted in 100% larval mortality in *Ae. aegypti, Cx. quinquefasciatus*	[26]
20E agonist methoxyfenozide (larvae)	*C. morsitans*	Premature moulting High larval mortality Incomplete pupation Adult females that survived had reduced fecundity/fertility	[104]
20E agonist halofenozide (larvae)	*Cx. pipiens*	Reduced number of cuticular hydrocarbons in larvae	[174]
20E agonist halofenozide (larvae)	*Cx. pipiens*	Failure to ecdyse Increased cuticular thickness	[175]
20E agonists Tebufenozide, methoxyfenozide, halofenozide & KU-106 (larvae)	*An. gambiae*	Larval mortality	[27]
Feeding larvae with transgenic algae expressing dsRNA against *HR3*^a^	*Ae. aegypti*	Larvae died prematurely Shorter larvae (body length) Abnormal midgut morphology Delay in life-cycle	[176]
***20E signaling regulates mating, fecundity and longevity***			
20E injection (virgin females)	*An. gambiae*	Lifetime refractoriness to mating in females Eggs laid were sterile	[25]
20E agonist methoxyfenozide (adult females)	*An. gambiae*	Reduction in mated females by 65% Reduced longevity	[23]
***20E signaling regulates mosquitoes’ ecdysteroid levels***			
Reducing 20E titer by silencing* spook* (adult females)	*An. gambiae*	Decreased ecdysteroid production in ovaries	[44]
Reducing 20E activity by injecting E220^b^	*An. gambiae*	Fourfold decreased ecdysteroid production 26 h after blood meal	[105]
***20E signaling regulates Plasmodium*** ***and bacterial infection***			
20E agonist methoxyfenozide (adult females)	*An. gambiae*	Infection by *Plasmodium falciparum* reduced by 87%	[23]
20E agonist halofenozide (adult females)	*An. gambiae*	*P. berghei* infection prevalence reduced by ~ 40% Reduction in oocyst intensity	[24]
20E agonist halofenozide (USP-silenced adult females)	*An. gambiae*	No effect on *P. berghei* infection prevalence	[24]
EcR silencing (adult females)	*An. gambiae*	Reduced *P. falciparum* oocyst prevalence by 11–24% Reduced* P. falciparum *extrinsic incubation period, as indicated by earlier invasion of salivary glands with sporozoites Higher infectious sporozoite prevalence and intensity in EcR-silenced females at 10 dpi and 12 dpi, respectively	[105]
Reducing 20E activity by injecting E220^a^ (adult females)	*An. gambiae*	Reduced oocyst intensity Reduced *P. falciparum* EIP, as indicated by earlier invasion of salivary glands with sporozoites	[105]
20E injection 24 h before infection (adult females)	*An. gambiae*	Reduced *P. berghei* oocyst prevalence and intensity Reduced *E. coli* infection	[106]
20E injection 2 h after infection (adult females)	*An. gambiae*	No effect on *P. berghei* oocyst prevalence and intensity	[164]
20E injection (adult females)	*An. coluzzii*	*P. falciparum* oocyst prevalence increased by ~ 93% *P. falciparum* oocyst intensity increased by > 100%	[125]
***20E signaling regulates pyrethroid resistance***			
Reducing 20E titer by silencing* spookiest*^c^ (adult females)	*Cx. pipiens pallens*	The resistant strain became increasingly susceptible to deltamethrin	[107]
Reducing 20E titer by silencing shade^c^ (adult females)	*Cx. pipiens pallens*	The resistant strain became increasingly susceptible to deltamethrin	[108]

*Ae*.,* Aedes*;* An*.,* Anopheles*;* Cx*.,* Culex*; dpi, days post-inoculation; EcR, ecdysone receptor; 20E, 20-hydroxyecdysone; USP, ultraspiracle protein

**Fig. 1:**
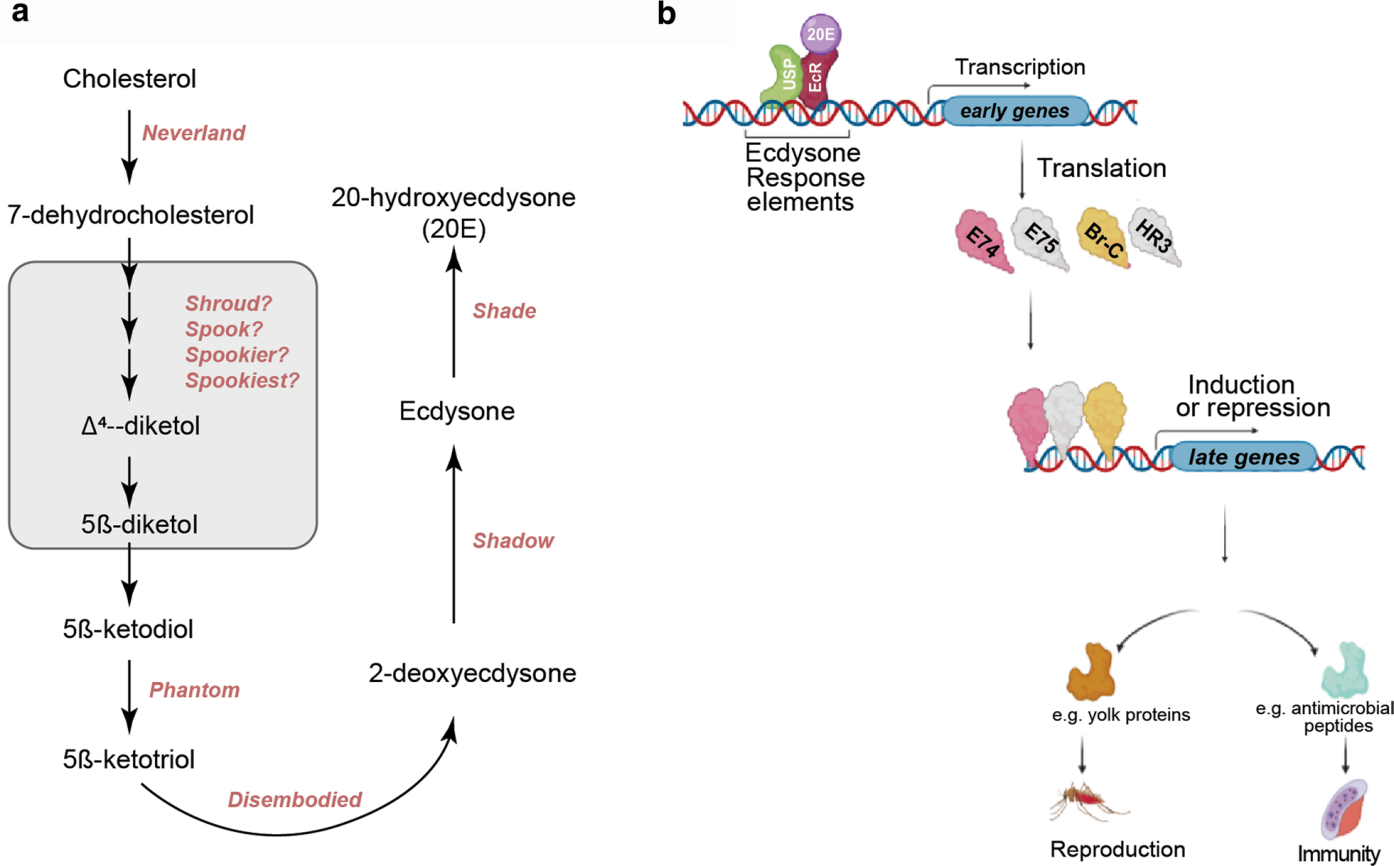
20-Hydroxyecdysone (*20E*) signaling in insects. **a** 20E biosynthesis from dietary cholesterol, based on studies in *Drosophila melanogaster*. Metabolites and enzymes are indicated in black and pink, respectively. The “black box” (where the exact metabolites/enzymes are unknown) is indicated by the grey area. Orthologues of these enzymes have been characterized in mosquitoes. **b** Once 20E binds to its heterodimer EcR/USP receptor, the latter is activated and acts as a transcription factor, binding to an enhancer region known as the ecdysone response elements (EcRE). Binding of EcR/USP to EcRE activates the transcription of early genes (*E75*, *E74*, *HR3* and *Broad-Complex*). These four early genes in turn act as transcription factors, inducing or repressing the expression of downstream genes involved in vector competence and vector abundance. *Br-C* Broad complex, *EcR* ecdysone receptor, *USP* ultraspiracle

^a^*HR3* is one of the “early genes” in the 20E signaling cascade; see Fig. 1b

^b^E220, or ecdysone 22-oxidase, reduces 20E activity by converting the C22 hydroxyl group into a carbonyl group [177]

^c^*Spookiest* and* shade* code for cytochrome P450 enzymes involved in 20E biosynthesis; see Fig. 1a

## An overview of the 20E signaling pathway in mosquitoes

### 20E biosynthesis is a multi-enzyme process

From the food they ingest, mosquitoes obtain cholesterol, the precursor molecule for 20E biosynthesis [28, 29]. In larvae and pupae, the conversion of cholesterol to 20E takes place in the prothoracic glands [30, 31], while in adults 20E biosynthesis occurs in the ovaries and fat body (females) and in the the accessory glands (males) [32–35]. Although most knowledge related to 20E biosynthesis comes from studies in *Drosophila*, orthologues of the enzymes involved in this process have been characterized in mosquitoes. The first enzyme in this process, neverland, catalyzes the conversion of dietary cholesterol to 7-dehydrocholesterol [36–38], which is in turn metabolized to 5ß-ketodiol* via* Δ^4^-diketol, 5β-diketol and a few uncharacterized intermediate metabolites (Fig. 1a). Hence, the term “black box” has been used to describe this part of the 20E biosynthesis pathway [37]. Nonetheless, research has shed some light on the intermediate steps and characterized the enzymes, spook, shroud, spookier and spookiest in the *Drosophila melanogaster* model [39–42]. Of these, spook and shroud orthologues have been identified in *Ae. aegypti* and/or *An. gambiae* [38, 43]. In particular, *spook* knockdown by RNA interference in *An. gambiae* decreased the production of 20E in the ovaries, confirming that spook has the same function in both *Drosophila* and *An. gambiae* [44]. The metabolite 5β-ketodiol is further converted to 5β-ketotriol and then transformed to 2-deoxyecdysone before it is finally changed to ecdysone (Fig. 1a); these three steps are catalyzed by cytochrome P450 (CYP) enzymes CYP306a1 (*phantom*), CYP302a1 (*disembodied*) and CYP315A1 (*shadow*), respectively [45–48]. Finally, ecdysone is secreted from the prothoracic glands or ovaries into the hemolymph. It then enters the fat body where it is oxidized to the active form 20E, by another cytochrome p450 enzyme, namely CYP314a1 (shade) [49]. 20E is then release from the fat body and transported to different cells and tissues, as needed. Orthologues of these four enzymes have been identified and functionally characterized in *An. gambiae*, confirming their roles in 20E biosynthesis in the mosquito [32].

### Activation and regulation of the 20E signaling cascade

In mosquitoes, the 20E signaling pathway is activated when 20E binds to its nuclear receptor, the ecdysone receptor complex (Fig. 1b). The ecdysone receptor complex is a heterodimer consisting of the ultraspiracle protein (USP) and the ecdysone receptor protein (EcR) (Fig. 1b). USP and EcR are orthologues of the mammalian retinoid-X receptor (RXR) and farnesoid X receptor (FXR), respectively [50, 51]. Both EcR and USP are members of the steroid receptor superfamily, which is characterized by five domains: A/B (transactivation), C (DNA-binding), D (hinge), E (ligand-binding) and F (transactivation) [52, 53]. Recently, the F-domain of *Ae. aegypti* EcR was shown to bind to the metal ions Cu^2+^ and Zn^2+^, thereby inducing a helical structure in the protein and promoting ligand binding specificity [54, 55]. Only EcR binds to 20E, but a study in *Drosophila* revealed that EcR requires heterodimerization with USP to successfully bind the hormone [56]. It has also been reported that USP might be involved in the allosteric regulation of EcR, altering its conformation to favor the hormone- and DNA-binding properties of EcR [57, 58]. The EcR–USP complex acts as a transcription factor, binding with high affinity to the ecdysone response elements (EcRE) [59], an enhancer located in the upstream regulatory regions of ecdysone-responsive genes. In *Ae. aegypti*, EcRE are composed of DNA motifs that are either inverted or direct repeats [60]. Binding of EcR–USP to EcRE activates the transcription of “early genes” such as *E75*, *E74*, *HR3* and *Broad-Complex* [61–67]. These early genes in turn also act as transcription factors, inducing or repressing the expression of several downstream genes which control reproduction, immunity and development (Fig. 1b) [68]. However, there are some cases where the EcR–USP transcription factor directly binds to the EcRE regions of downstream genes, such as the vitellogenin gene [69].

Two EcR isoforms (A and B) have been identified in *Ae. aegypti*, and they appear to vary in biological function as well as spatial and/or temporal expression [63, 70–72]. For example, in the fat body, EcR-A expression increases during the vitellogenic period from 12 h post-blood meal (hPBM) to 24 hPBM, and then decreases by 36 hPBM; while EcR-B is most abundant in the pre-vitellogenic and post-vitellogenic period [72]. Similarly, two USP isoforms have been described in *Ae. aegypti* [53, 71, 73]. The abundance of USP-A in the fat body is highest in the pre-vitellogenic and late vitellogenic period, while USP-B is highly expressed during vitellogenesis [73]. In the midgut of *Ae. aegypti* larvae, the EcR-B and USP-A isoforms are more abundant than EcR-A and USP-B [74]. In addition to the mosquito fat body and midgut, isoforms of the ecdysone receptor subunits have also been detected in the ovaries and male accessory glands [32, 70]. Also, while there is currently no experimental evidence in mosquitoes, to our knowledge, EcR isoforms have also been detected in the central nervous system of *Agrotis ipsilon* [75], *Apis mellifera* [76] and *Bombyx mori* [77].

Several regulators coordinate the spatio-temporal expression and activation of EcR and USP. For example, before a blood meal, USP is bound to the nuclear factor HR38 in the fat body of *Ae. aegypti*, but HR38 is later displaced by EcR during the vitellogenesis period (12–24 hPBM) [78]. Another important regulator is the “early gene” *E75*. Three isoforms of E75 (E75A, E75B, and E75C) have been detected in the fat body of *Ae. aegypti* post-blood meal, and functional studies have revealed that silencing either E75A or E75C shifts the peak expression of EcR-A (which normally occurs 12–24 hPBM) to 24–30 hPBM [79]. Two additional proteins, FISC and βFTZ-F1, act as co-activators of EcR/USP in the fat body of blood-fed *Ae. aegypti* females, by recruiting and binding to the EcR/USP complex at the EcRE region of the *vitellogenin* promoter. This association was absent in the non-bloodfed cohorts [80]. Besides vitellogenesis, the timely regulation of metamorphosis also requires the presence cofactors to regulate EcR/USP. For example, in *Ae. aegypti* fourth instar larvae, the CREB-binding protein (CBP)—whose primary function is to loosen the chromatin structure to render the DNA regulatory regions accessible to transcription factors—suppresses the expression of EcR-A, to prevent premature molting. When CBP is silenced, *EcR*-*A* expression is elevated, and the larvae prematurely metamorphosed into pupae [63, 81].

## 20E signaling regulates multiple physiological processes at different stages of the mosquito life-cycle

**Fig. 2:**
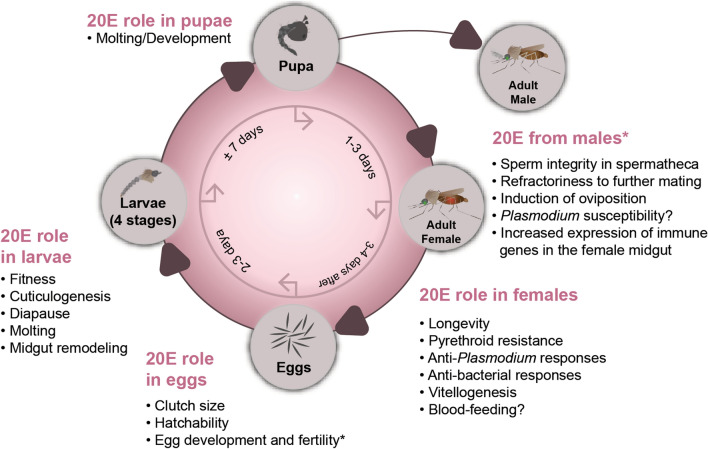
Manipulating 20E titers, activity or signaling affects several physiological processes at each stage of a mosquito life-cycle. Only processes that have been experimentally confirmed in mosquitoes are represented. The asterisk (*) indicates that this is not the role of 20E in males, but rather the role that the male-secreted 20E plays in females, once it is transferred to their atrium during mating

Depending on the environmental conditions, mosquito eggs hatch into larvae within 2–3 days (reviewed in [82, 83]). The newly emerged larvae then undergo four successive molts from first to fourth instar larvae, lasting in total approximately 5–10 days, prior to becoming pupae. About 1–3 days later, adult mosquitoes emerge from their pupal cuticle. The 20E signaling pathway is an integral part of mosquitoes’ life-cycle (Fig. 2) as it regulates several physiological processes associated with development, reproduction or susceptibility to pathogen infection, as discussed below.

### Egg development and oviposition

#### Egg development requires nutrients from blood

**Fig. 3:**
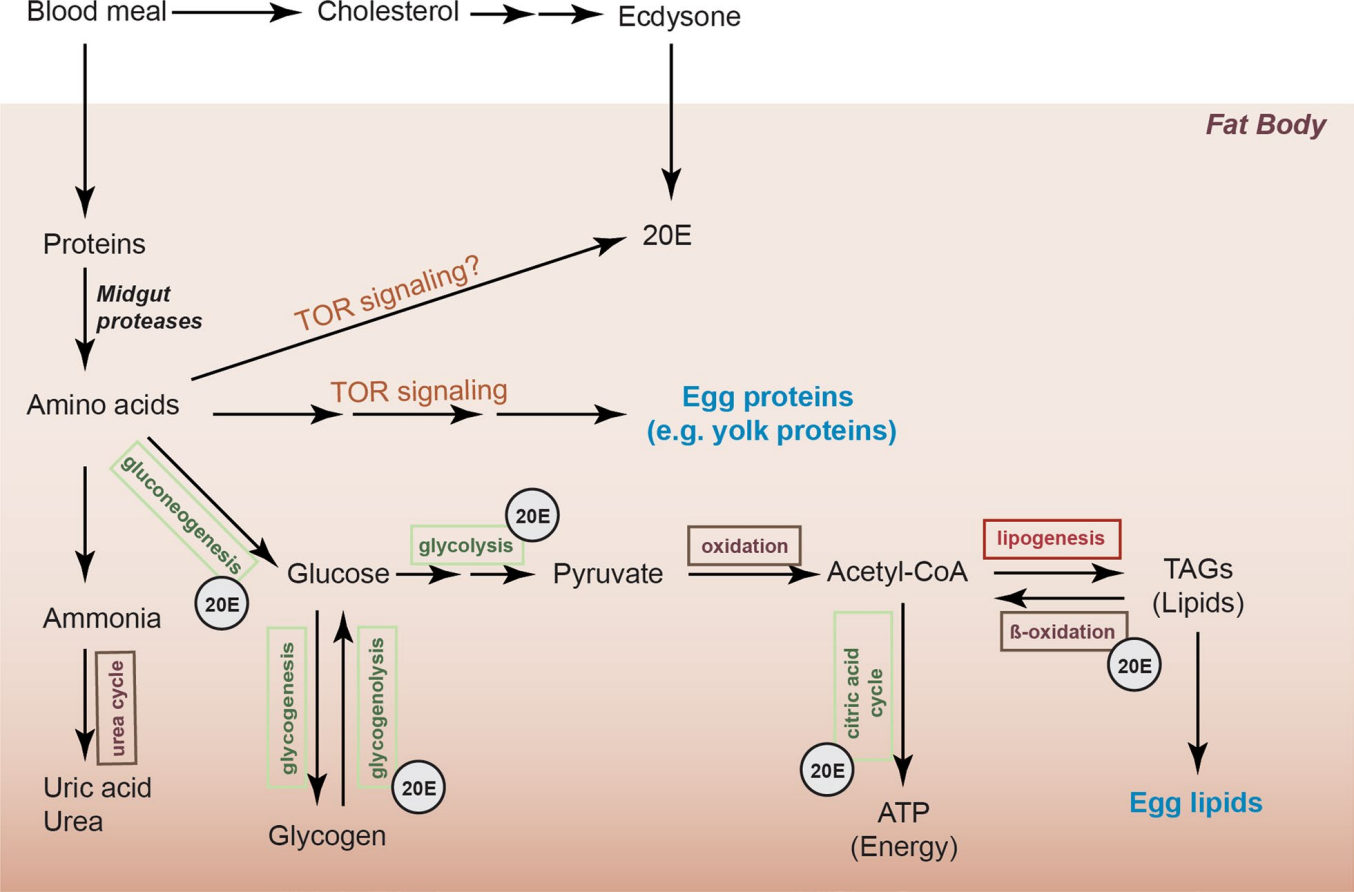
Anautogenous mosquitoes use the blood nutrients to produce egg components in the fat body. In *Aedes aegypti*, digestion of the blood meal involves several metabolic processes (indicated in boxes), many of which are regulated by 20E signaling, as indicated by the “20E” label. The carbohydrate-related metabolic pathways are indicated in green boxes, while the lipid-related metabolic pathways are indicated in red boxes.* CoA* Coenzyme A,* TAGs* triacylglycerols,* TOR* target of rapamycin

In anautogenous female mosquitoes, egg development requires nutrients obtained from a blood meal. After the ingested blood is digested to release cholesterol and proteins (Fig. 3), the cholesterol is used for 20E production, while the midgut proteases hydrolyze the proteins into amino acids. These amino acids are incorporated into various metabolic pathways in the fat body such as lipid and carbohydrate metabolic pathways, resulting in the production of lipids, yolk proteins and energy (Fig. 3) [84]. The pathways for carbohydrate metabolism, including glycogen metabolism, gluconeogenesis, the citric acid cycle and glycolysis, have previously been found to be upregulated at 18–24 hPBM, which is also the peak of 20E synthesis in females [85]. Further analysis revealed that silencing EcR downregulated the expression of several genes involved in glycolysis and glycogen metabolism, resulting in an increase in fat body glycogen, decreased ATP levels, and the accumulation of sugars (glucose and fructose) [85]. Dong et al. [86] later reported that 20E regulates carbohydrate metabolism* via* the HR38 nuclear transcription factor. Similarly, 20E signaling was also shown to regulate lipid metabolism in the fat body (Fig. 3), as silencing of EcR resulted in increased levels of triacylglycerols and decreased β-oxidation [87]. This allows the insect to store lipids as either an energy source for egg maturation or to incorporate these lipids in the developing oocytes [88].

#### 20E-mediated oviposition requires brain-secreted hormones

**Fig. 4:**
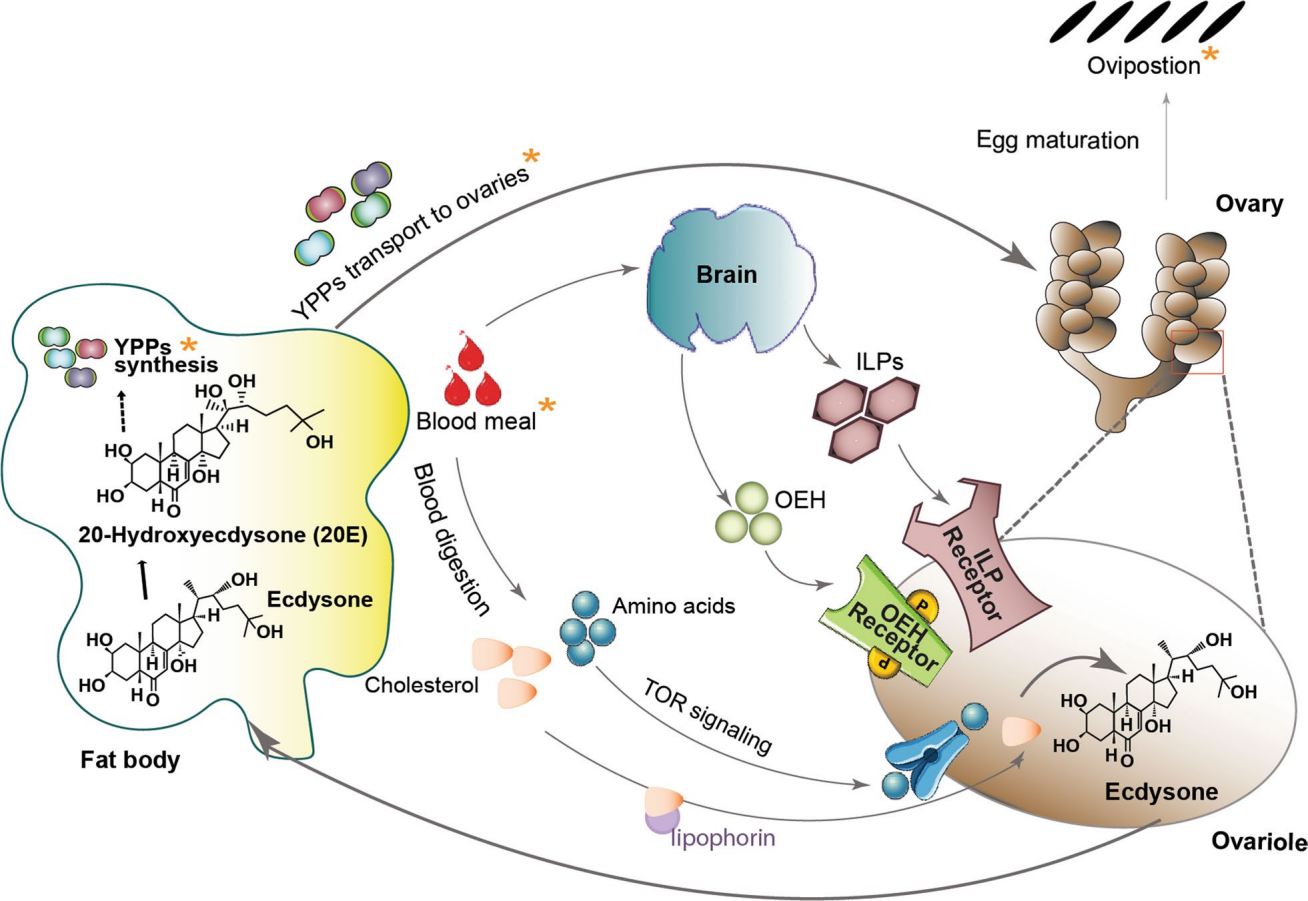
20E signaling regulates oogenesis (for details see text). The steps regulated by 20E signaling in mosquitoes are indicated by orange asterisks (*)* OEH* Ovary ecdysteroidogenic hormone,* ILP* insulin-like peptide, *YPPs* yolk protein precursors

In *Ae. aegypti*, the ingestion of a blood meal triggers the brain to release two neurohormones—ovary ecdysteroidogenic hormone (OEH) and insulin-like peptide 3 (ILP3) — into the hemolymph [89, 90] (Fig. 4). ILP3 and OEH bind to their receptors (the insulin and OEH receptors, respectively), located on the follicle cells of the ovarioles [91, 92]. This binding triggers a phosphorylation cascade, which in turn activates the target of rapamycin (TOR) and insulin pathways, and ultimately blocks the activity of the glycogen synthase kinase 3 (GSK3) protein [93]. Blocking of GSK3 results in the proliferation of follicle cells, an indication that the ovarioles are ready to produce ecdysone [93]. Hence, the blood-derived cholesterol (transported by lipophorin, a carrier protein synthesized in the fat body) and amino acids (*via* amino acid transporters) enter the follicle cells where they serve as building blocks for ecdysone synthesis [94–98]. Ecdysone is then released from the ovaries and enters the fat body where it is converted into 20E (Fig. 4). In the fat body, 20E triggers the synthesis of yolk protein precursors (YPPs) such as vitellogenin, vitellogenin carboxypeptidase or cathepsin b-like protease [35]. These YPPs are released into the hemolymph and transported to the growing oocytes in the ovaries where they are taken up by receptor-mediated endocytosis (Fig. 4). Although the regulation of YPP transport has not been investigated in mosquitoes, Carney et al. [99] reported that *Drosophila* females with EcR mutations displayed decreased transport of YPPs to the ovaries compared to untreated controls. The oocytes, now fully developed into eggs, are laid by mosquitoes in aquatic environments.

### Larvae

Mosquito larvae undergo four developmental stages, from the first instar to the fourth instar, all of which take place in aquatic environments. 20E signaling is essential to molting from one larval stage to the next, as indicated by the high levels of 20E and its receptor in *Ae. aegypti* during larval ecdysis [63, 100, 101]. In addition, it has been reported that manipulating 20E titers and signaling in *Culex quinquefasciatus*, *Cx. pipiens*, *Ae. aegypti*, and *Anopheles gambiae* impairs larval fitness, development, survival, cuticulogenesis and molting (Table 1).

### Pupae

Key events in mosquito pupal development include sexual dimorphism [102], programmed cell death and cell differentiation [71], ecdysis [63] and the formation of adult structures such as wings [103]. In *Ae aegypti*, both male and female pupae display an increase in titers of 20E, ecdysone, 2-deoxyecdysone and other steroid molting hormones. The ecdysteroid titers reach a much higher level in males than in females, and the peak appears earlier in male pupae than in female ones [102]. This difference may explain why males eclose into adults sooner than females, an important feature for mating success in adult mosquitoes [102]. In terms of programmed cell death and cell differentiation, Parthasarathy and Palli [71] observed that during the initial pupal stages in *Ae. aegypti*, both EcR isoforms (EcR-A and EcR-B) are present in larval cells undergoing apoptosis, while EcR-B is present in the imaginal diploid cells of pupae, indicating that both isoforms facilitate the turnover of larval cells while EcR-B plays an additional role in the development of imaginal cells. The role of 20E in the formation of wing structure has not yet been investigated in mosquitoes; however, in the domesticated silkworm *Bombyx mori*, manipulation of 20E titers suggests a pivotal role in healthy wing development [103]. Collectively, the roles of 20E signaling in different aspects of pupal development are in agreement with the observation that manipulating 20E signaling in the tsetse fly *Glossina morsitans* results in incomplete pupation [104].

### Adults

#### Female adults

In female adults, several physiological parameters are affected by manipulating 20E signaling, including susceptibility to *Plasmodium* infection [105, 106], longevity [23], insecticide resistance [107, 108], blood-feeding and vitellogenesis [34, 52, 64, 109] (Fig. 4). As vitellogenesis has been discussed in above (section Egg development and oviposition), in this section we focus on the other phenotypes.

The sporogonic cycle of malaria parasites begins when *Anopheles* mosquitoes ingest *Plasmodium* gametocytes while feeding on infected hosts [110]. In the insect midgut, these gametocytes rapidly differentiate into male and female gametes. The zygotes that are formed from fertilization develop into motile ookinetes which, upon crossing the midgut epithelium and its basal membrane, transform into oocysts that remain fixed at the interface of the midgut and hemolymph [111]. Once fully matured (~ 14 hPBM), the oocyst “bursts” and releases sporozoites in the hemolymph [112]. These navigate to the salivary glands where they are ready to be injected into the next host during a following blood meal [111]. For the development of transmission-blocking interventions, three parameters related to the sporogonic cycle are relevant: (i) oocyst prevalence, which refers to the percentage of mosquitoes with contain oocysts after taking an infectious blood meal [113]; (ii) oocyst intensity, which is the average number of oocysts per mosquito; these are measured as functions of transmission-reducing activity and transmission blocking activity respectively, with TBA considered to be a more useful assessment of efficacy given that even just one oocyst can produce many infectious sporozoites [114]; (iii) duration of the sporogonic cycle, known as the extrinsic incubation period (EIP), which is a measure of the time needed for a mosquito to become infectious [115]. The EIP can be affected by factors such as environmental temperature and by underlying genetic features of both the vector and parasite [115].

Manipulating the titers, activity or signaling of 20E affects the parasite’s oocyst prevalence (*Plasmodium falciparum* and *P. berghei*), oocyst intensity (*P. falciparum* and *P. berghei*), and EIP (*P. falciparum*) [105, 106]. However, the molecular mechanisms by which these parasite parameters are regulated are poorly understood. From an immune response perspective, it is possible that 20E signaling regulates *P. berghei* development* via* several immune effectors, including antimicrobial peptides, prophenoloxidases, CLIP serine proteases or lysozymes [106]. In addition, given the increase in the number of phagocytic cells and activity after 20E injection [106], it is possible that the 20E pathway reduces susceptibility to *P. berghei* infection by increasing the phagocytic defense mechanism.

In terms of longevity, boosting 20E signaling by applying methoxyfenozide (see section 20E agonists) onto the thorax of *Anopheles* mosquitoes resulted in a reduced lifespan with increasing concentrations of methoxyfenozide [23]. This is important because if the mosquito lifespan becomes shorter than the parasite EIP, malaria transmission is effectively reduced (reviewed in [116]). In the context of insecticide resistance, previous reports have shown that silencing *spookiest* or *shade* in deltamethrin-resistant *Cx. pipiens* mosquitoes rendered these mosquitoes more susceptible to the pyrethroid [107, 108]. To date, there is no report, to our knowledge, directly linking 20E biosynthesis enzymes to *Anopheles* resistance to insecticides. However, previous studies have shown that *shade* is overexpressed in dichlorodiphenyltrichloroethane (DDT)-resistant *An. gambiae* [117], while *shade* and *phantom* are overexpressed in DDT- and pyrethroid-resistant *An. funestus* [118], suggesting that these may also be implicated in insecticide resistance in *Anopheles* spp. Nonetheless, functional studies will be required to directly assess the role of *shade* and *phantom* in insecticide resistance in *An. gambiae* and/or *An. funestus*. Moreover, it may be relevant to determine whether the overexpression of 20E-related genes in insecticide-resistant *An. gambiae* affects their longevity, as it could have implications for the parasite EIP, and thus malaria transmission. Finally, it is interesting to note that 20E also plays a role in the extent of nutrient uptake, a feature that has been observed in different insects (e.g. *An. freeborni*, *Helicoverpa armigera*, and *Bombyx mori*) injected with 20E [109, 119, 120]. While it appears that 20E only plays an indirect role in nutrient seeking by blocking dopamine signaling which normally promotes food-seeking behavior [119, 121], this observation is yet to be investigated in mosquitoes.

#### Males

In *An. gambiae* males, 20E is exclusively synthesized in the male accessory glands (MAGs), and its production increases from the day of adult emergence until the male become sexually mature and active (i.e. 3–6 days post-emergence) [32]. During copulation, some of the male-synthesized 20E is transferred to the female atrium, as part of a mating plug secreted by MAGs, and it is replenished in the male after copulation [32]. This male-to-female transfer of 20E has been observed in at least four anopheline species, including *An. gambiae*, *An*. *arabiensis*, *An. stephensi* and *An. dirus* [122]. Once in the female atrium, the male-derived 20E regulates several processes, such as oviposition, egg fertility and refractoriness to further copulation, and it helps maintain the integrity of the sperm in the spermatheca [25, 123–125]. Although little is known about the mechanisms whereby the male-derived 20E regulates these processes, it has been reported that the female atrium-specific MISO protein interacts with the male-derived 20E to regulate egg production [123]. Overall, both male- and female-synthesized 20E contributes towards the reproductive behavior and success of anopheline female mosquitoes (Fig. 2).

## The potential of chemical control interventions targeting 20E signaling

### 20E agonists

20-hydroxyecdysone agonists are insect growth regulators (IGRs) that compete with 20E to bind to its EcR receptor complex, thereby overactivating the 20E signaling pathway. Interestingly, both EcR and USP subunits of the receptor complex are needed for successful 20E agonist activity [24, 126]. The most studied class of 20E agonists are the dibenzoylhydrazine (DBH) compounds [126, 127]. Currently, many IGRs based on DBH compounds are commercially available, such as tebufenozide (RH-5992), methoxyfenozide (RH-2485), halofenozide (RH-0345), fufenozide, chromafenozide (ANS-118) or RH-5849 [128–130]. These compounds were initially formulated against lepidopteran and coleopteran crop pests; however there is increasing evidence that they could also be used to control mosquito populations at different developmental stages, including eggs, larvae and adults [23, 26, 27, 131].

Water treatment with methoxyfenozide has been shown to reduce egg hatch rate in *Cx. pipiens* [131], as well as larval mortality in *An. gambiae*, *Ae. aegypti*, and *Cx. quinquefasciatus* [26, 27]. The effect of 20E agonists on pupae is yet to be tested in mosquitoes, but treating *Spodoptera litura* pupae with RH-5849 resulted in pupal development abnormalities and a subsequent decrease in adult emergence [132]. In *An. gambiae* adults, it has been demonstrated that methoxyfenozide and halofenozide reduced *P. falciparum* and *P. berghei* transmission, respectively [23, 24]. In addition, fecundity, fertility, mating success and adult longevity were all significantly decreased after DBH exposure [23]. As such, DBH compounds affect both vector abundance and vector competence and have the additional benefit of showing minimal effect on non-target species, as opposed to conventional insecticides which may be toxic to humans and other arthropods (Table 2).

**Table 2:** Insecticidal properties of the dibenzoylhydrazine compounds which have shown promising results against mosquitoes

Dibenzoylhydrazine compounds	Cross-resistance	Absence of cross-resistance	Toxicity	Off-targets
Methoxyfenozide	Organophosphate [145, 178, 179] - Chlorpyriphos (low) - Azinphosmethyl Pyrethroids [180] - Deltamethrin Others [145, 180, 181] - Cyromazine (low) - Fipronil (low) - Abamectin - Teflubenzuron (low)	Pyrethroid [145] - Bifenthrin Organochlorine [182] - Indoxacarb Others [145] - Spinosad	Mammals, birds and fish (very low)	Organisms [130, 183] Earthworms Birds (low) Fish (low) Honey bees Agricultural pests
Tebufenozide	Dibenzoylhydrazines [133] - Methoxyfenozide Pyrethroids [133] - Deltamethrin (low) Organophosphate [178, 179] - Azinphosmethyl Others [133, 134] - Abamectin - JS118	Pyrethroid - Cypermethrin Organophosphate - Trichlorfon - Phoxim - Acephate Others Fipronil Chlorfenapyr	Mammals, birds and fish (very low)	Agricultural lepidopteran pests
Halofenozide	n/d	n/d	Mammals, birds and fish (very low)	None against fish *Gambusia affinis* [184]
RH-5849	n/d	n/d	Damage to DNA of human blood lymphocytes [185]	*Daphnia magna* [186]

n/d, No data were available

Resistance to DBH compounds has been studied in the lepidopterans *Plutella xylostella*, *Cydia pomonella*, and *Spodoptera exigua* [133–138], and two mechanisms have been identified. As with classic insecticides, the first mechanism involves an increase in the activity of detoxification enzymes such as carboxylesterase, aryl-acylamidase, cytochrome P450s or glutathione-*S*-transferases [138–141]. While an increased expression of cytochrome P450s also constitutes the resistance mechanism of some carbamates, pyrethroids and organochlorines (reviewed in [142]), it is interesting to note that cross-resistance between DBH compounds and these classic insecticides is not always guaranteed (see following paragraph). The second resistance mechanism, identified in *P. xylostella*, involves the microRNA *miR-189942*, which decreases the expression of the EcR-B isoform, thereby reducing the susceptibility to fufenozide (because fewer binding sites are available for the 20E agonist) [143]. However, resistance to DBH compounds is unstable due to fitness costs such as higher mortality rate and decreased reproductive capacity that are associated with the DBH-resistant phenotype [137, 138, 144, 145]. As such, most insects revert back to the susceptible phenotype when the 20E agonist is removed [137, 138]. This is a significant advantage over classical insecticides where long-term use has resulted in fixed population-wide genetic changes that confer resistance. Alternatively, the emergence and spread of DBH resistance could be delayed by including available synergists to the DBH formulations [146], such as metyrapone and diethylmaleate, which inhibit the activities of oxidative and glutathione-*S*-transferase enzymes, respectively [146].

Another important consideration before implementing a chemical control strategy based on 20E agonists is the phenomenon of cross-resistance. This occurs when insects are resistant to multiple insecticides because the insecticides share similar modes of action [147]. Studies in lepidopterans with methoxyfenozide and tebufenozide suggest that cross-resistance between DBH compounds is highly likely (Table 2), while cross-resistance between a DBH compound and the currently WHO-approved classes of insecticides (pyrethroids, organophosphates, carbamates, and organochlorines) is insecticide dependent. For example, while cross-resistance is observed between methoxyfenozide and deltamethrin, no such link is observed between methoxyfenozide and bifenthrin, although both deltamethrin and bifenthrin are pyrethroids (Table 2). In mosquitoes, cross-resistance between methoxyfenozide, pyrethroids, organochlorines (DDT) and carbamates has been characterized [148]. The authors of that study found that *Anopheles* populations which were resistant to DDT, carbamates and pyrethroids (regardless of the mechanism of pyrethroid resistance) were still susceptible to methoxyfenozide [148]. Collectively, these findings suggest that malaria vectors could be effectively controlled by a rotational strategy between DBHs and conventional insecticides, as part of an insecticide resistance management plan. Such a plan could, for example, involve (i) a rotation between pyrethroids and DBHs (with or without synergists/antimalarials) on LLINs, or (ii) a rotation between DBHs (with or without synergists/antimalarials), pyrethroids, DDT and carbamates for IRS and larvicides.

Another limitation of LLINs and IRS interventions is that they are designed to target mosquitoes indoors. Therefore, exophilic and exophagic vectors, such as *An. arabiensis*, are poorly controlled by these approaches [149]. To overcome this challenge, attractive toxic sugar baits (ATSB) have been proposed. The components of ATSB include a floral scent, a sugar solution and an oral insecticide, with the aim to attract, feed and kill mosquitoes, respectively [150, 151]. This technique has already been proven successful against *An. gambiae* and *An. arabiensis* populations in experimental trials [152, 153], and it would thus be worth investigating if the addition of DBH compounds to ATSB could enhance their efficacy. Additionally, one could also target exophilic/exophagic mosquitoes at the immature aquatic stages using DBH compounds (with or without synergists) as larvicides and ovicides (Table 1).

### IGRs that reduce 20E titers and signaling

The 20E signaling pathway is also targeted by IGRs that interfere with its activity or reduce 20E titers. These include cucurbitacins (triterpenoid class of natural products) [154, 155], chlorantraniliprole (CAP; ryanoid class of pesticides) [156, 157] and clothianidin (neonicotinoid class of pesticides) [158]. CAP targets the insect calcium channels to deplete intracellular calcium [156, 157], while clothianidin targets the acetylcholine receptor and affects the insect immune system [158]. With respect to 20E biosynthesis and 20E signaling, a study in *Drosophila* showed that cucurbitacins are able to either displace a steroid hormone bound to EcR or prevent the formation of the EcR/USP heterodimer complex [154]. Application of CAP on *Chilo suppressalis* was shown to reduce vitellogenin expression and 20E titers and to increase the expression of three 20E biosynthetic enzymes (phantom, spook and shade), likely in response to the decrease in 20E levels [157, 159]. Finally, a study of the effect of clothianidin on *Aphis gossypii* revealed that this insecticide reduced vitellogenin and EcR expression [158].

As expected for insecticides that target 20E signaling, the phenotypes induced by these chemicals include impaired development (*P. xylostella*, *B. mori, C. suppressalis*), reduced fecundity (*P. xylostella*,* C. suppressalis*,* A. gossypii*) and mortality (*P. xylostella*,* C. suppressalis*,* A. gossypii*) [156–160]. However, despite these promising phenotypes, IGRs interfering with the 20E pathway may not be suitable for use against *Anopheles* vectors for multiple reasons. Firstly, unlike 20E agonists, clothianidin may be toxic to humans, rendering it unusable for public health [161]. Second, while 20E agonists have minimal effects on non-target species [162], CAP has shown adverse effects on honeybees, even at sublethal doses, and as such shows off-target effects on other important insects [163]. Third, Werling et al. [105] showed that a reduction in 20E signaling accelerates the *P. falciparum* sporogonic cycle in such a way that mosquitoes are able to transmit malaria sooner. Therefore, chemical control interventions targeting 20E signaling should rather focus on overactivating the pathway, as do the DBH compounds.

## Concluding remarks

The development of novel interventions is urgently needed to counteract insecticide resistance in malaria vectors. In this review, we have summarized the importance of 20E throughout the mosquito life-cycle and consolidated some of the experimental evidence that supports the use of 20E agonists as part of an integrated approach to malaria vector control. Not only are 20E agonists already commercially available, but results from preliminary laboratory experiments suggest that they are effective against all mosquito life-stages (Table 1), with minimal toxicity to non-target species (Table 2). The efficacy of 20E agonists is mainly attributed to their ability to overactivate the 20E signaling pathway, a biological process which regulates vector abundance and competence in mosquitoes.

While the molecular mechanisms by which 20E signaling regulates mosquito reproduction and fecundity have been extensively studied, more research is needed to elucidate how 20E signaling (and 20E agonists) regulates *Anopheles*’ susceptibility to *Plasmodium* infection. From an experimental perspective, the timing of 20E injection (either before or after *Plasmodium* infection) influences whether or not the parasite’s sporogonic cycle is affected. Indeed, injection of 20E in *An. gambiae* 24 h prior to infection was found to result in a decrease in *P. berghei* oocyst prevalence and intensity [106], while there was no effect on these parameters when the injection occurred shortly after infection [164]. These divergent outcomes may result from a difference in the timing of 20E-regulated immune priming [106], and this would be worthwhile investigating.

Second, it is likely that DBH compounds (i.e. non-steroid 20E agonists) and 20E regulate *Plasmodium* development by distinct mechanisms, although they both bind to EcR. For example, even though exposure of *An. gambiae* to halofenozide and 20E both decrease *P. berghei* oocyst prevalence and intensity, the authors of these studies observed that only 20E induced the expression of immune genes [24, 106], therefore leaving unanswered the question of what could be the potential non-immune mechanisms by which 20E agonists regulate *P. berghei* competence. Possibilities include that DBH compounds regulate epigenetic modifications [165, 166], the formation of the peritrophic matrix [167], the expression of specific midgut factors that are essential for *Plasmodium* invasion [112], metabolism [142], signaling pathways (e.g. c-Jun N-terminal kinase [JNK] pathway), or all of these simultaneously. Clarifying these issues will help researchers to determine the full impact of 20E agonists on *Anopheles* vector competence.

Third, it is still unclear whether the male-derived 20E contributes to *Anopheles* susceptibility to *P. falciparum* (NF54 strain) infection. Dahalan et al. [125] showed that mating increased both *P. falciparum* oocyst prevalence and intensity in *An. coluzzii*. This effect was attributed to the male-to-female transfer of 20E, since 20E injection of virgin *An. coluzzii* produced similar results [125]. On the other hand, Marcenac et al. [168] reported no effect on parasite prevalence or intensity in mated *An. gambiae* and *An*. *stephensi*. Therefore, it is possible that the male-derived 20E is not solely responsible for the phenotypes observed but, rather, it acts in conjunction with other female genes that are affected by post-mating. Consistent with this notion, 13 genes (7 in the lower reproductive tract and 6 in the carcass) were found to be differentially expressed in females after mating in *An. coluzzii versus* *An. gambiae* [169]. Further studies should investigate whether one of these genes is responsible for the discrepancy observed between the findings of Dahalan et al. [125] and Marcenacet al. [168].

While we have presented vector competence and vector abundance as two separate entities that are each individually regulated by 20E through distinct mechanisms, in reality, both *Plasmodium* sporogony and vitellogenesis/egg production occur simultaneously in mosquitoes (reviewed in [170]). Moreover, the two processes are positively correlated in *P. falciparum*-infected *An. gambiae* mosquitoes [105]. This implies that the genes regulating anti-*Plasmodium* responses and those regulating fecundity are coordinately expressed after a mosquito takes an infected blood meal, and possibly co-regulated. Could it be that 20E acts as a “master regulator” which coordinates the timeous expression of genes involved in fecundity or immunity? If so, this further reinforces the premise discussed in this review that 20E is a worth-investigating target for the chemical control of malaria vectors.

## Data Availability

Not applicable.

## Abbreviations

WHO: World Health Organization
EcR: Ecdysone receptor
20E: 20-hydroxyecdysone
hPBM: Hours post-blood meal
YPP: Yolk protein precursor
DBH: Dibenzoylhydrazine
JNK: c-Jun N-terminal kinase pathway
TOR: Target of rapamycin
